# Recent Developments in Nanomaterials-Modified Membranes for Improved Membrane Distillation Performance

**DOI:** 10.3390/membranes10070140

**Published:** 2020-07-03

**Authors:** Saikat Sinha Ray, Harshdeep Singh Bakshi, Raghav Dangayach, Randeep Singh, Chinmoy Kanti Deb, Mahesh Ganesapillai, Shiao-Shing Chen, Mihir Kumar Purkait

**Affiliations:** 1 Institute of Environmental Engineering and Management, National Taipei University of Technology, Taipei City 106, Taiwan; harsh.d.singh777@gmail.com (H.S.B.); raghavdangayach9@gmail.com (R.D.); randeep@iitg.ac.in (R.S.); 2 School of Chemical Engineering, Vellore Institute of Technology (VIT), Vellore 632014, India; chinmoykanti.deb@gmail.com; 3 Department of Chemical Engineering, Indian Institute of Technology, Guwahati 781039, India; mihir@iitg.ac.in

**Keywords:** membrane distillation, membranes, desalination, nanomaterials, fouling, wetting

## Abstract

Membrane distillation (MD) is a thermally induced membrane separation process that utilizes vapor pressure variance to permeate the more volatile constituent, typically water as vapor, across a hydrophobic membrane and rejects the less volatile components of the feed. Permeate flux decline, membrane fouling, and wetting are some serious challenges faced in MD operations. Thus, in recent years, various studies have been carried out on the modification of these MD membranes by incorporating nanomaterials to overcome these challenges and significantly improve the performance of these membranes. This review provides a comprehensive evaluation of the incorporation of new generation nanomaterials such as quantum dots, metalloids and metal oxide-based nanoparticles, metal organic frameworks (MOFs), and carbon-based nanomaterials in the MD membrane. The desired characteristics of the membrane for MD operations, such as a higher liquid entry pressure (LEPw), permeability, porosity, hydrophobicity, chemical stability, thermal conductivity, and mechanical strength, have been thoroughly discussed. Additionally, methodologies adopted for the incorporation of nanomaterials in these membranes, including surface grafting, plasma polymerization, interfacial polymerization, dip coating, and the efficacy of these modified membranes in various MD operations along with their applications are addressed. Further, the current challenges in modifying MD membranes using nanomaterials along with prominent future aspects have been systematically elaborated.

## 1. Introduction

The changing environment and depleting sources of water lead to increased demand for clean and drinking water for domestic, agricultural, and industrial use. According to the National Ocean Service-US, around 97 percent of Earth’s water is in the ocean, but due to its ultra-high salinity, this water is unsuitable for direct use. In this context, membrane distillation (MD) is highly effective and efficient to desalinate ultra-high saline waters. Hence, the MD separation method offers various combinations to be used in desalination, wastewater treatment, pharmaceutical, food, and biomedical applications for different applications. The lower membrane mechanical strength, temperature, and pressure requirements distinguish MD from other membrane separation processes [1]. In addition, MD can be easily combined with various processes and renewable or alternative sources of energy, making it not only energy-efficient but also effective in separation. These properties make MD a desired membrane separation process for large-scale applications. As earlier mentioned, MD is a thermally induced membrane separation process widely utilized for desalination and wastewater treatment [1,2,3,4]. In addition, MD separation is a non-isothermal process in which a hot feed side and a cold permeate side are separated by a thin hydrophobic membrane. The temperature difference between feed and permeate results in a state of thermodynamic disequilibrium, which leads to a vapor pressure difference. Under the presence of this driving force, vapor molecules are transported from the feed side to the permeate side and condense as a pure distillate [5]. Membrane distillation can be used in different arrangements, such as air gap membrane distillation (AGMD), direct contact membrane distillation (DCMD), sweeping gas membrane distillation (SGMD), and vacuum membrane distillation (VMD) [4,5]. These different types of MD processes carry their own advantages and can be used accordingly in relation to the application type and demand. MD membranes, as mentioned earlier, face numerous challenges such as membrane fouling, surface wetting, and low permeate flux resulting in comprehensive work to address these challenges by integrating nanoparticles (NPs) in these MD membranes. These include quantum dots, metal-based NPs (titania (TiO_2_), silver (Ag)), silica-based NPs, metal organic frameworks (MOF), and carbon nanotubes (CNTs) that are used as either surface modifiers or blended in the polymeric membrane matrix. Nanomaterials are often used to improve membrane flux and salt rejection, enhance surface hydrophobicity, mitigate membrane fouling, and enhance the physical and chemical characteristics of the membrane to ensure superior functionality [6]. Therefore, research has focused on the engineering of superhydrophobic, anti-wetting surfaces, including the development of MD membranes with new generation nanomaterials for enhanced MD performance.

### 1.1. Timeline of MD Membranes

**Figure 1:**
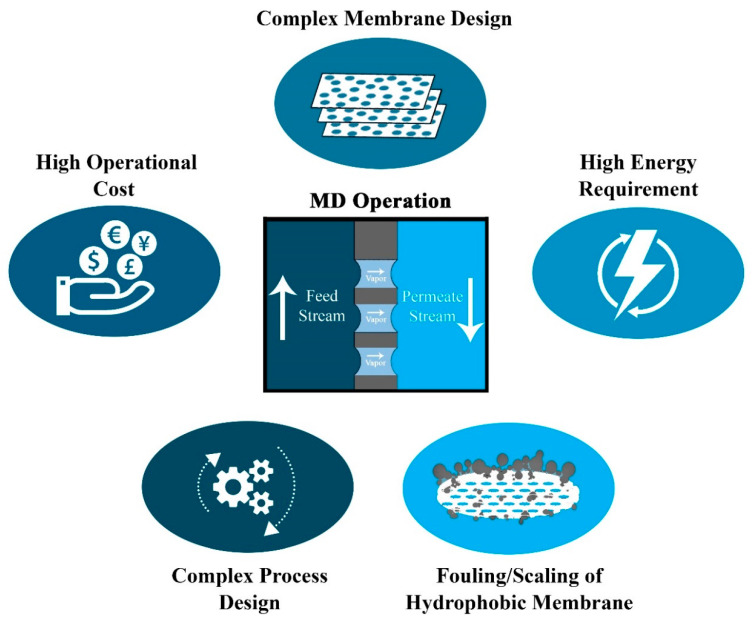
Challenges of an ideal membrane distillation (MD) process in the field of water treatment and desalination.

Membrane distillation has emerged as an effectual process for desalinating salty water under low temperature and pressure conditions [7], unlike other methods, such as nanofiltration and reverse osmosis, that demand high energy, hence substantial capital investment [8,9]. Despite advancements, the popularity of the MD process is declining due to the lack of availability of appropriate membranes due to various challenges in the MD operation (Figure 1) that makes it difficult to come up with an ideal MD membrane separation process compared with other membrane separation processes. The first MD patent came in the year 1963 [10] and the first research publication in 1967. Later, in the 1960s, the first set for asymmetric membranes was developed for membrane desalination applications, which was revolutionary in the field of separation technology for the next five decades [1,2,3,11]. However, there is still scope for advancement and development in the field of MD membranes in order to propose the successful application of the MD process on an industrial scale. The most important aspect of using the MD process for large-scale applications is the integration of MD with other processes and utilizing a cheaper source of energy to make it a cost-effective, eco-friendly, and efficient process. The operational cost of the membrane distillation system much depends on the source of energy, where conventional heat sources such as burning coal, petroleum, or electricity make the MD process very expensive (10.8 USD/m^3^ for AGMD). The operational cost of MD processes can be significantly reduced (2.68 USD/m^3^ for AGDM) if an alternative, cheaper heat source, such as waste heat and solar heat is utilized, making its operational cost comparable to conventional desalination technologies [11].

Remarkably, MD operations also have a higher rejection of micro-pollutants when compared with both nanofiltration (NF) and reverse osmosis (RO) processes [12]. Although MD operations can be energy-efficient and have a higher rejection percentage, membrane fouling and wetting are major concerns affecting the efficiency of the process [13,14]. Extensive research on the modification of polymeric membranes by the incorporation of nanoparticles (NPs) is being conducted in order to mitigate these concerns. NPs are ultrafine particles with at least one dimension less than 100 nm. These ultrafine particles attribute very different physicochemical characteristics compared with their bulk counterparts, despite being made of the same materials, and hence the mean particle size and size distribution play a vital role in the applications of these NPs [15,16].

**Figure 2:**
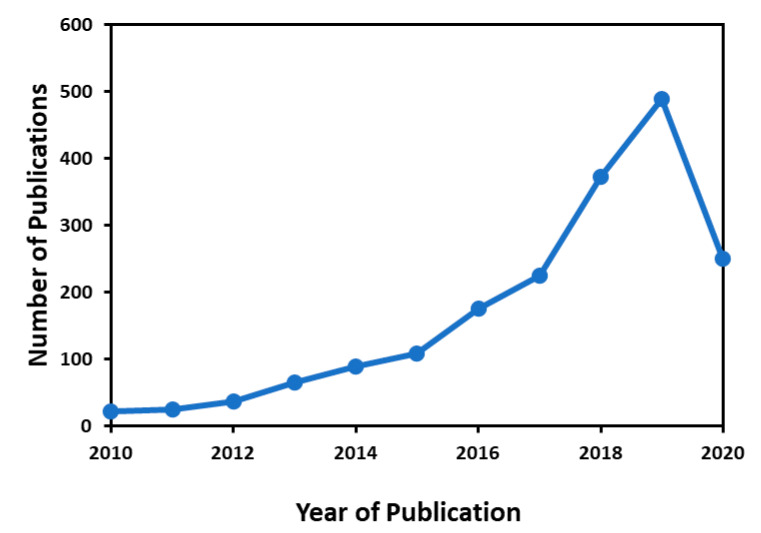
Survey on the number of published research papers since 2010. Database acquired from the advanced Scopus scholar search system with the terms “membrane distillation” and “nanomaterials” as in April 2020.

A careful survey was executed to show the progressive development in the field of MD membranes in the last decade, as shown in Figure 2, in terms of research publications related to “membrane distillation” and “nanomaterials”. The database was taken from an advanced scholarly search system based on Scopus. Therefore, it can be concluded that nanomaterial-based MD membranes have gained a lot of attention in recent desalination research and development.

Although there are many advantages to MD, the progress of MD and MD membranes on a commercial scale has been literally slow due to various challenges faced, as pointed out in Figure 1. Although there are many advantages to MD, the progress of MD and MD membranes on a commercial scale has been literally slow. The progress in the first three decades was minimal, and most of the studies were basic in nature, focusing on the understanding of the MD process and its different configurations. Later in the emerging phase, many research and development sectors came along to tackle the serious issues related to the wetting and fouling of MD membranes.

### 1.2. New Generation Nanomaterials-based MD Membrane

Over the past decade, nanomaterials such as metalloid and metal NPs, MOFs, CNTs, QDs, and graphene and its derivatives have attracted a lot of attention of researchers and thus have emerged as pioneer candidates for potential materials to be used for MD membrane modification. These nanomaterials have been used for various purposes to mitigate membrane fouling and pore wetting as they improve the physicochemical properties of the membrane [17].

**Figure 3:**
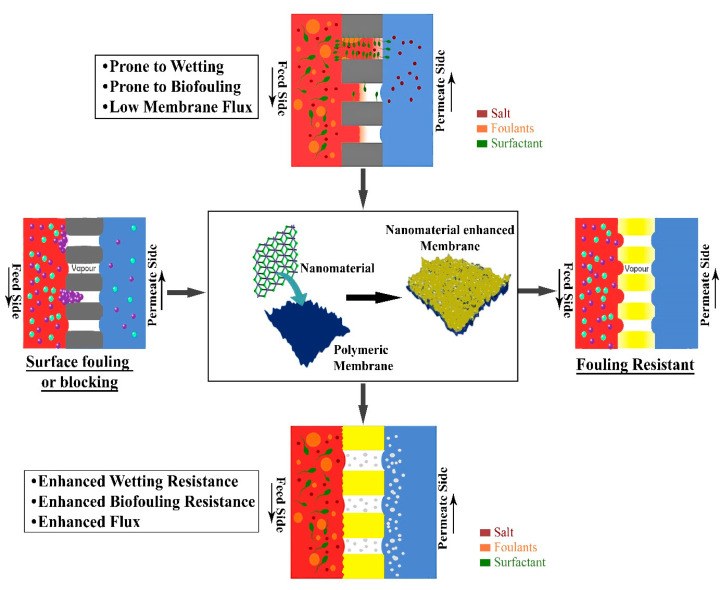
Effectiveness of nanomaterials on the MD membrane.

Metallic NPs possess a hierarchal structure with multilevel roughness, thus improving the hydrophobic character of the membrane [18]. Metallic NPs are often used to generate an uneven surface to the membrane surface. This uneven surface naturally leads to the formation of air pockets, which allow for improper contact between the water and the membrane surface, resulting in the improvement of the hydrophobic properties of the membrane [19,20]. Further, some of the NPs, such as Ag, TiO_2,_ or SiO_2,_ possess antibacterial characteristics due to their ability to react with the thiol (-SH) group present in the cell wall of microorganisms which prevents biofilm formation [21]. MOFs, on the other hand, are one of the most common fillers that are used in mixed matrix membranes. Additionally, the unique physical and chemical properties of MOFs make them the most commonly used fillers in mixed matrix membranes. The organic ligands of MOFs show strong attraction towards the polymeric chains, describing excellent filler properties [22]. Further, MOFs-based membranes exhibit tailorable pore structure and size, which makes them application-specific in terms of selectivity and permeation [23]. They possess an ordered arrangement with high pore volume and specific surface area, which modifies the membrane structure resulting in improved transmembrane flux [24]. Carbon-based nanomaterials are a superior class of nanomaterials possessing novel characteristics like a large surface area to volume ratio, high mechanical strength, and tendency to reduce fouling propensity, which makes them attractive materials to fabricate multifunctional composite membranes [25]. Quantum dots have recently attracted a lot of attention as an MD membrane modifier due to their low cost of production and highly desirable physicochemical properties [26]. Figure 3 shows the efficacy of new generation nanomaterials on the MD membrane. 

New generation NPs used in MD studies have been critically reviewed in this investigation with a focus on the enhancement of the anti-fouling and superhydrophobic properties of the membrane. Modified membranes have been evaluated using characteristics such as pure water flux, salt rejection, pore size and porosity, water contact angle, liquid entry pressure (LEPw) value, and long-term operation. Further, we have thoroughly discussed membrane modification methods, which usually are neglected while discussing about MD membrane fabrication.

The main goal of this review was to give a comprehensive outlook on the various state-of-the-art membranes fabricated for MD application using novel materials such as metallic NPs, CNTs, graphene and its derivatives, and MOFs. Even though literature focused on the improvement of membrane characteristics using an NP addition has been published before, our study aims at giving detailed scientific discussions on the mechanisms which govern the improved properties of nanoparticle-modified membranes [27,28,29,30]. This review article focuses on the effectiveness and strategies used to design membrane nanostructures for improved performance in MD. In addition, this review article compares the advantages and disadvantages of emerging NPs and nanomaterial-based membranes. Finally, the research gap and problems associated with nanomaterial-based membranes in previous MD investigations have been addressed with potential solutions to fabricate these membranes for real-world applications.

## 2. Fundamentals of MD Membrane

Lippman’s non-isothermal liquid separation was first introduced in 1907 [31]. Subsequently, Aubert carried out a detailed investigation into this process [32]. This phenomenon of non-isothermal separation was called thermo-osmosis due to the nature of the liquid separation. As mentioned above, the separation of liquids takes place in the form of vapor in MD. Therefore, theoretically, any type of membrane, dense or porous, charged or neutral, or hydrophilic or hydrophobic, in nature may be used. Porous and hydrophobic membranes, however, are commonly used for better results, as described later in this section. In addition, the material used for the synthesis of MD membranes is selected on the basis of the properties to be imparted to the membranes [33,34,35]. Therefore, this section gives details about the various developments and advancements of the MD membrane. In later sections, commonly used membrane modifications via the incorporation of nanomaterials for the development of specific membranes for the MD process are discussed.

### 2.1. Membrane Materials and Structure

**Figure 4:**
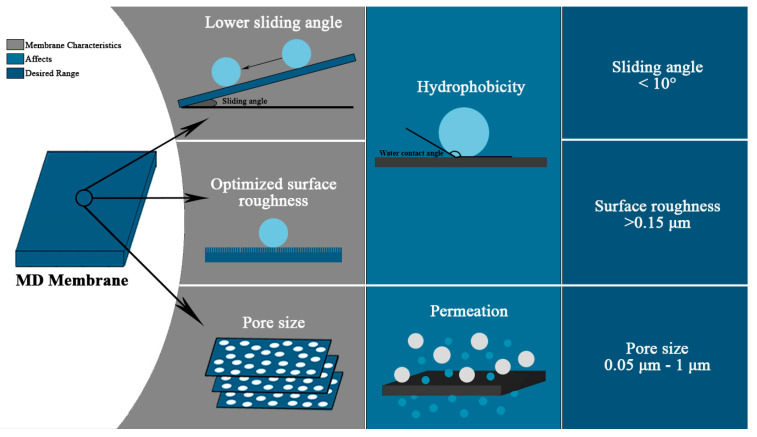
Basic membrane characteristics and their corresponding impacts that affect MD performance.

The material used for fabricating the membrane is of extreme importance when designing a typical MD system because it affects both the heat and the transfer of mass across the membrane, which influences the long-term performance of the membrane [36]. Materials like polytetrafluoroethylene (PTFE), polyvinylidene fluoride (PVDF), and polypropylene (PP) are the most widely used polymers for MD membrane fabrication due to their intrinsic hydrophobic nature and good processability. A more hydrophobic surface would prevent the penetration of the liquid phase into the membrane pores, due to the surface tension forces, while allowing the vapor molecules to pass through the porous surface. Membrane porosity and thickness also vary along with the pore size, which could lead to a different conclusion on the overall effect on MD performance. It is believed that the properties such as the thickness, tortuosity, pore size, and porosity dictate the resistance to mass transfer in the MD process [37]. Figure 4 shows the basic membrane features, along with the desired range that affects the performance of MD.

Apart from the choice of the polymer, the concentration of the polymer in the dope solution can affect the resultant morphology and performance of the membrane. In a recent study, the effects of the dope concentration of polyethersulfone (PES) on the morphology and average pore size of the achieved membrane were studied. It was observed that with an increase in the concentration of PES in the spinning dope solution, the average pore size of the PES hollow fiber substrate decreased [38,39]. Therefore, a key area of scientific research focuses on the design, modification, and manufacture of hydrophobic membranes for MD processes. Nanomaterials were widely used as additives to enhance the structure of the membranes and achieve desired characteristics [40]. Some primary nanomaterial properties include superior chemical and thermal stability, high surface area to volume ratio, and exceptional mechanical resistance. Consequently, nanomaterial doping can lead to a major improvement of the membrane structure as it increases porosity, modifies membrane morphology, and also enhances surface hydrophobicity [41]. SiO_2_-NPs doping in PVDF led to the formation of an asymmetric arrangement with a porous sponge layer as well as a finger-like macro void layer, which resulted in improved VMD performance [42]. The incorporation of superhydrophobic alumina NPs in nanofibrous PVDF membranes improved the liquid entry pressure (LEPw) and water contact angle of the membrane structure, which helped in the treatment of highly concentrated brines in an AGMD operation [43]. Water vapor transport improved in graphene oxide-modified PTFE membranes due to the selective sorption of water onto the epoxy, hydroxyl, and carboxyl groups in GO [44]. A carbon nanotube-immobilized PP membrane showed a greater mass transfer coefficient than the pristine PP membrane, which was enabled in the separation of pure water from saline water [45]. Thus, different kinds of nanomaterials can be used to fabricate multifunctional membranes possessing novel properties, which will improve MD performance.

### 2.2. MD Membrane Perquisites

An MD system operation is heavily dependent upon the membrane’s intrinsic properties. It is essential to have a perfect blend of structural and physicochemical characteristics to achieve optimum performance. The characterization of different properties like surface morphology, membrane thickness, pore size, and geometry sheds light on the physical characteristics of the material used for manufacturing. On the other hand, contact angle, sliding angle, surface roughness, and LEPw measurements provide insight into the membrane’s hydrophobicity. The membrane should have low mass transfer resistance and thermal conductivity, while also maintaining high thermal and chemical stability for the performance required [46]. Table 1 provides an outline of the desired characteristics required in membranes that are currently used for the MD application.

### 2.3. MD Membrane Modification

Membranes for MD operations are generally manufactured using phase inversion (solution casting) and electrospinning methods, which are subsequently modified with dip coating, graft polymerization, and interfacial polymerization [56]. The phase inversion technique involves defusing/de-mixing, where under optimal conditions, a homogeneous polymeric solution is transformed into a membrane. This technique can be further classified according to the methods used for transformation, viz., immersion precipitation, thermally induced phase separation, and precipitation by controlled evaporation [2]. Thermally induced phase separation conduces to the formation of the membrane when the parent polymeric solution is de-mixed under high-temperature conditions. In the immersion precipitation method, the membrane is developed by the interchange of the solvent between the polymeric solution and coagulation solvent where the polymeric solution is immersed. In contrast, the membrane is formed during evaporative phase inversion by the volatilization of the solvent used to prepare the initial polymeric solution [57,58].

**Figure 5:**
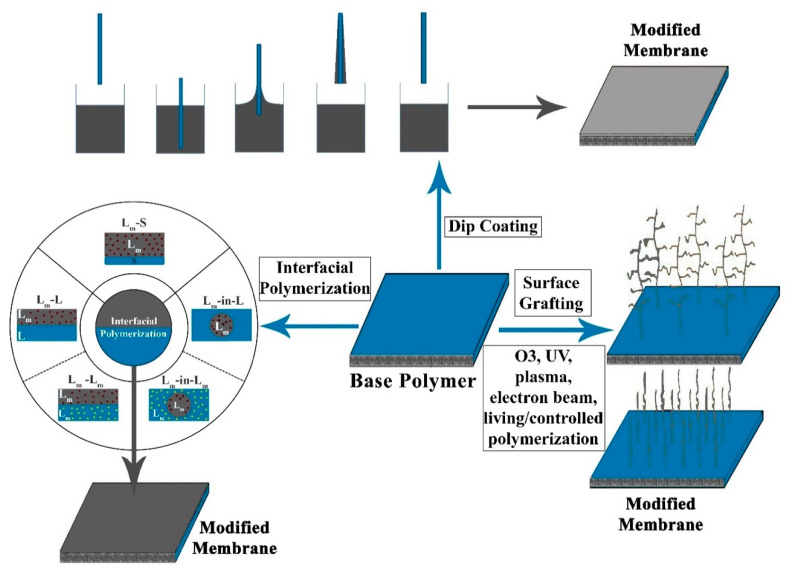
Methodologies for the incorporation of nanomaterials onto an MD membrane.

The electrospinning technique, which is a variation of the electro-spraying process, involves a polymeric solution or melt subjection to high electrical fields, which reduce the polymeric blend surface tension [59,60,61,62]. Under this condition, the polymer is stretched and diluted, resulting in the formation of nanofibers collected on a fixed surface [63]. Different methods, including surface coating, surface grafting, plasma polymerization, interfacial polymerization, and dip coating are used to incorporate NPs in the MD membrane to enhance their properties, enabling them to overcome challenges such as fouling, wetting, fluxing, and porosity by providing them with the necessary characteristics. Figure 5 indicates the schematic diagram of various methodologies for the incorporation or doping of nanomaterials in the MD membrane.

#### 2.3.1. Surface Grafting

Surface grafting is a chemical treatment in which the membrane material chains are activated and then grafted by chemical reaction or intense radiation. These grafted macromolecular chains form covalent bonds with the surface of the membrane. These bonds established on the MD membrane surface guarantee long-term stability and prevent surface delamination [64]. In contrast to physical modification, surface grafting forms a covalent linkage between the polymeric surface of the membrane and the molecules of the nanomaterial used as a modifier; this is generally carried out in two different ways, i.e., grafting-to and grafting-from.

In the grafting-to technique, the end-functionalized active molecules react with the surface of the membrane, while in grafting-from, free radical sites are generated by the base polymer, which reacts with the modifiers to form a modified surface. Comparing these two methods, grafting-to is more convenient to control as, in this case, the pre-synthesized reactive groups can be purified before grafting, whereas grafting-from provides an edge when it comes to controlling the thickness of the grafted layer, as in this case the modifiers can be added over time. Further, in the grafting-from method, various processes are available to activate the surface of the polymeric membrane for grafting polymerization, which includes plasma treatment, UV irradiation, ozone treatment, and gamma-ray irradiation [65]. Many recent studies have shown that surface grafting can be used to modify a hydrophilic membrane surface into a hydrophobic surface, making them suitable for MD operations. In a recent study, a hydrophobic yttria-stabilized zirconia (YSZ) hollow fiber membrane was obtained by grafting fluoroalkylsilanes (FASs) on the pristine membrane surface. The achieved modified membrane attributed a high contact angle (up to 140°), high mechanical strength, and considerable chemical and thermal stability [66]. In another study, grafting induced by direct radiation was used by El-Arnaoty et.al., for the incorporation of ZnO NPs on the polyamide membrane surface to enhance its anti-biofouling properties [67].

#### 2.3.2. Plasma Polymerization

Plasma polymerization involves electrically induced atomic disintegration of monomers that give rise to different active particles. The generated activated particles combine with the surface to form a highly branched and crosslinked structure. This deposited layer ensures a lower mass transfer coefficient and decreases the water flux of the MD membrane. Plasma polymers show stronger adhesion to the substrate along with higher thermal stability. Plasma polymerization is a one-step method and hence provides a greater advantage over other modification techniques by cutting down several steps. Further, this incorporated layer only has a thickness of 1 to 2 µm and has very minimal impact on the porosity of the MD membrane compared with other techniques. This modification technique is widely utilized in the incorporation of metal/metal oxide nanomaterials on the membrane surface, and in a recent study, silicon dioxide particles were grown on a PVDF membrane surface to enhance its stability and reduce the flux decrease [68]. Similar to this, in various other studies, the incorporation of different metal/metal oxide nanomaterials was carried out using plasma polymerization to enhance the physicochemical properties of the membrane surface without compromising its porosity. Moreover, this method of MD membrane modification is environment-friendly [69].

#### 2.3.3. Interfacial Polymerization

Interfacial polymerization is a step-growth technique in which polymerization takes place at the interface between two non-miscible phases providing them with specific chemical and topological properties, like anisotropic shapes, hollow structures, and alternative surface chemistry [70]. The fabrication of a polymer material by interfacial polymerization includes two major aspects, which are developing an interface between the two non-miscible phases and distributing the monomers in these two phases. These interfaces can be further categorized as liquid–liquid emulsion (L–L), liquid–solid emulsion (L–S), and liquid in liquid emulsion (L–in–L). The factors affecting interfacial polymerization include temperature, humidity, and purity of the reactants. This technique has been used for the surface enhancement of membranes by incorporating carbon, graphene, metal/metal oxide nanomaterials, and their composites. In the latest study carried out by [71], graphene-oxide-TiO_2_ nanofillers were incorporated onto PA membranes by interfacial polymerization, providing them with higher water vapor permeance and selectivity.

#### 2.3.4. Dip Coating

Dip coating is one of the straightest techniques available to incorporate NPs onto an MD membrane [72]. The process is carried out in three steps: (a) immersion and dwell time, (b) deposition, and (c) evaporation of the solvent. The base polymer is kept perpendicularly in the solution containing NPs until these modifiers properly settle on the surface, after which, the membrane is withdrawn out of the solution and is allowed to dry. In this same manner, commercially available polyester (PET) membranes were hydrophobically modified by PDMS-coated SiO_2_ NPs yielding a chemically stable, superhydrophobic membrane (dip-PET) [73]. The coating thickness, structural integrity, and pore size of the post-modification membrane are dependent on several process parameters like dipping time, the concentration of the dipping polymer, and concentration of the crosslinking solution [74].

### 2.4. State of the Art of Nanomaterials Doped MD Membrane

These specialized MD membranes achieved better results both in terms of water flux and the salt rejection rates compared with the pristine membrane, as mentioned earlier. Doping certain nanomaterials transforms the chemical aspect of the membrane; therefore, it helps to achieve better performance in the MD operation. A short summary of various nanomaterials incorporated in membranes for various MD applications is demonstrated in Table 2.

## 3. Incorporation of Nanomaterials for Enhanced Performance

Nanotechnology has played a crucial part in the development and advancement of membrane science. Nanotechnology has provided excellent materials, such as metallic NPs, carbon nanotubes, graphene, and metal organic frameworks, as membrane modifiers so as to achieve the required membrane functional and structural characteristics. These materials provided membranes with exceptional characteristics, such as selective permeability, chemical resistance, anti-fouling nature, mechanical strength, and thermal stability, resulting in better operational and functional properties of the membranes. The use of nanotechnology has contributed to the advancement of the membrane life span, which reduces the overall cost of a separation process. This makes the membrane separation processes competitive with other traditional processes and proposes their use in applications of a large scale.

**Figure 6:**
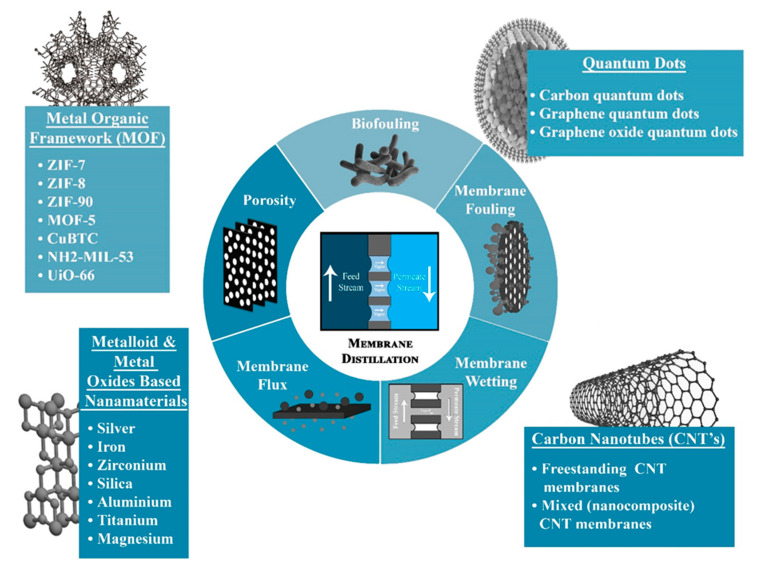
Recent advancements towards the application of MD in seawater desalination by utilizing various classes of new generation nanomaterials.

In addition to that, the incorporation of NPs as membrane modifiers to improve fouling resistance and minimize wettability is focused. Few have reported the utilization of silica-based NPs and TiO_2_-NPs for improved membrane hydrophobicity [75,77]. Therefore, the use and effects of different nanomaterials on membrane modifications are discussed in detail in this section. The section further discusses the various applications of modified membranes. As before, MD-based studies were virtually non-existent in the 1980s, but during the early 2000s, most of the developments and advances were directed towards the growth of the MD technique. The recent research boom has been observed in MD membranes where it is directed towards commercialization, with a particular emphasis on improvements in material and structure involving the use of different types of nanomaterials. Figure 6 shows the current evolution of MD membranes involving different groups of new generation nanomaterials for efficient MD output. 

### 3.1. Metalloid and Metal Oxides Based Nanoparticles

**Figure 7:**
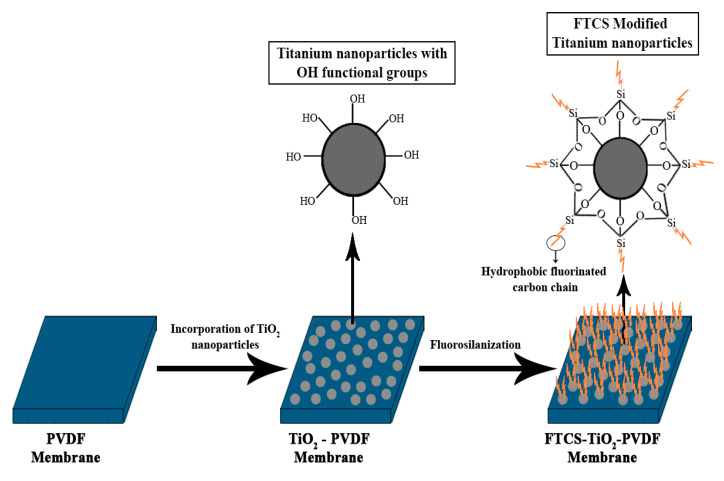
Schematic diagram for the superhydrophobic modification of FTCS-TiO2-PVDF membranes.

Metalloids and metal oxide NPs have the potential to produce synergistic effects when combined with different types of materials and hence are extensively used in enhancing the properties of MD membranes. In recent studies, various oxides-based NPs like SiO_2_, TiO_2_, ZnO, and Al_2_O_3_ were used to enhance the membrane flux and wetting resistance for MD operations [53,77]. Among these, TiO_2_ NPs have gained a lot more consideration due to their high availability, and excellent physicochemical, antibacterial, and anti-fouling properties [86,87]. TiO_2_ NPs are added to prevent fouling by rendering hydrophilicity to these hydrophobic MD membranes, reducing the surface interaction of these organic foulants. TiO_2_ NPs can be incorporated either by mixing them in bulk with the membrane substrate or by coating them over the surface. A superhydrophobic MD membrane was developed by coating a TiO_2_ nanoparticle on the surface of a polyvinylidene fluoride (PVDF) membrane, followed by fluorosilanization of the surface. The generated FTCS-TiO_2_-PVDF membrane had a significantly higher LEPw value (190 kPa) and contact angle (163 ± 3°). The incorporation of TiO_2_ NPs provided the surface with a hierarchical arrangement and -OH functional groups, ensuring uniform functionalization of perfluorododecyltrichlorosilane (FTCS). As illustrated in Figure 7, the incorporation of FTCS on the rough surface hydrolyzed the hydrophilic end on the TiO_2_ incorporated PVDF surface, exposing the hydrophobic fluorinated carbon chain [77]. In a similar manner, the FAS-PVDF-SiO_2_ nanofiber membrane was prepared, by the electrospinning of the PVDF-SiO_2_ solution, followed by fluorosilanization, by immersing the obtained PVDF-SiO_2_ nanofiber membrane into a fluoroalkylsilane (FAS)-containing solution. In this case, the achieved superhydrophobic surface featured an LEPw value of 195 kPa and a contact angle close to 160.5° [88]. In another study, a PVDF/SiO_2_ NPs composite membrane was developed by the phase inversion technique for a VMD operation. The incorporation of these superhydrophobic SiO_2_ NPs enhanced the hydrophobicity of the membrane. Though the composite membranes could not feature a superhydrophobic surface, they all showed higher hydrophobicity than the pristine PVDF membrane, with a maximum contact angle of 94°. The generated nano-composite membrane could achieve a maximum flux value of approximately 3 L m^−2^ h^−1^, 99.98% salt rejection rate, and an LEPw value greater than 3 bar, making it suitable for VMD operations for seawater desalination [42].

**Figure 8:**
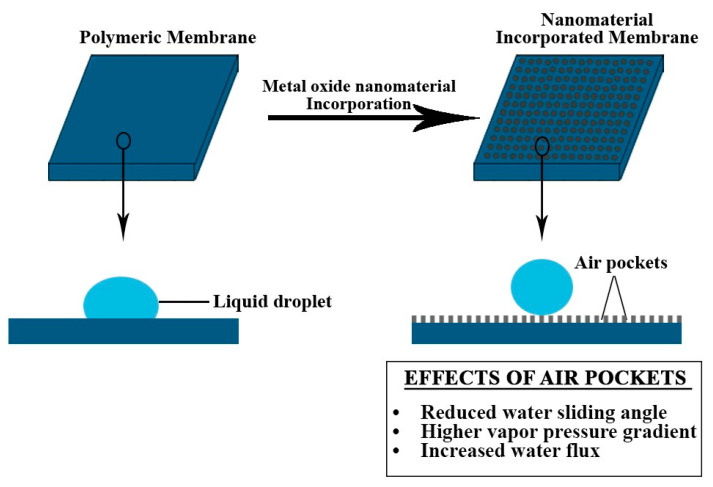
Illustration of air layer formation due to enhanced surface roughness.

The incorporation of metal oxide NPs on a polymeric MD membrane can significantly increase its surface roughness. In a study put forward by Dong Ma et al., the surface roughness of the developed FAS-PVDF-SiO_2_ membrane significantly raised from 92 to 218 nm as the weight percentage of the SiO_2_ nanomaterial in the dope solution increased from 0% to 8% [88]. This increase in surface roughness reduces the effective solid–liquid interface area along with an increase in the presence of air pockets, formed when the two layers collect a certain amount of vapor/air mixture. In the presence of external hydraulic pressure, these air pockets still keep deforming. They ensure that the water droplets move quickly and hence decrease the water sliding angles of the membrane. Further, the presence of pocket-like cells creates a resistance to heat transfer, which helps maintain a high temperature in the feed side, ensuring a higher vapor pressure gradient, thus increasing the water flux of the MD membrane [89,90]. Figure 8 demonstrates the effects of air pockets that result in superhydrophobicity and anti-wetting properties.

### 3.2. Carbon Materials

Carbon-based nanomaterials such as carbon nanotubes (CNTs), graphene, and graphene oxide are extensively utilized in membrane separation techniques due to their unique characteristics that enhance membrane permeability and selectivity [91]. Such nanomaterials allow water molecules to be transported swiftly and impart an anti-fouling character to the membrane, making them a successful candidate for MD technology. The following section illuminates the state-of-the-art MD membranes produced using these nanomaterials, with a focus on enhanced activity due to their addition.

#### 3.2.1. Carbon Nanotubes (CNTs)

Carbon nanotubes (CNTs) possess a wide range of superlative physical, chemical, and thermal characteristics, which make them a perfect filler material for polymeric membrane composites [92]. Carbon nanotubes are allotropes of carbon possessing a hexagonal lattice rolled up in a hollow cylindrical structure [93]. They have diameters ranging from 1 to 100 nm and up to a few millimeters in length, which may result in increased permeability within composite membranes [94]. CNTs are widely classified based on the number of sheet layers of graphene used for manufacture, i.e., single-walled CNTs (SWCNTs) and CNTs with multiple walls (MWCNTs) [95]. The introduction of CNTs offers alternative routes for transporting water vapor across the membrane, thereby dramatically enhancing the efficiency of the membrane [96]. Several CNT studies show that they improve the behavior of adsorption–desorption and reduce the frictional behavior of the walls, which provides favorable conditions for selective transmembrane flux [97].

CNTs also possess a nonpolar aromatic structure that is hydrophobic in nature and hence is used to improve and enhance the anti-wetting ability of the MD membrane, along with a significant contribution to enhance the mechanical strength, self-cleaning functions, and anti-fouling capacity of the MD membrane [25]. CNTs either form beads on the surface of the membrane, or they protrude out wholly which increases the microstructure roughness, contributing directly to surface hydrophobicity [97]. Silva et al., 2015, investigated the effects of MWCNT doping onto PVDF membranes and found an optimum 0.2 wt% that resulted in large-sized pores having a sponge-like consistency that resulted in a permeate flux of 9.5 × 10^−3^ L m^−2^ s^−1^ with a salt rejection of 100% over 60 min [98].

In another study, CNTs promoted the repulsion force required for Knudsen and molecular diffusion, enabled in the ease of surface diffusion, and deterred the boundary layer effect in viscous flow, allowing for increased vapor flux and anti-wetting characteristics [99]. CNTs in their bucky paper configuration also exhibit favorable properties such as high porosity (90%) and higher contact angle (113°) with low thermal conductivity. The CNT-bucky paper-based modified membranes seem to be equivalent to that of commercially available PTFE membranes. However, CNT-bucky paper-based membranes exhibit a lower lifespan and show flux decline in the MD operation due to micro-crack formation [100]. Thus, CNT-based membranes show interesting prospects in MD research, but a lot of investigation is pending regarding its viability.

#### 3.2.2. Graphene and Graphene Oxide (GO)

Graphene and graphene oxide (GO) are an emerging class of nanofiller materials used for MD application. They possess unique structural and functional properties that set them apart from traditionally used NPs [101]. Graphene modification leads to added hierarchal microstructure roughness that helps in fabricating robust anti-wetting surfaces. Furthermore, like CNTs, they also show selective permeability because of the various functional groups present on their surface. When graphene-based membranes were tested under a mixture of saline water containing various surfactants, they possessed exceptional anti-fouling characteristics, which are attributed to the charge neutrality of the fabricated membranes [102]. Woo et al. studied the effects of varying the graphene doping concentration onto PVDF and found that 0.5 wt% graphene had a superior contact angle, pore size, porosity, and LEPw as compared with neat membranes. However, increasing it further than 0.7 wt% resulted in the aggregation of graphene particles that was counterproductive for the AGMD operation [103].

**Figure 9:**
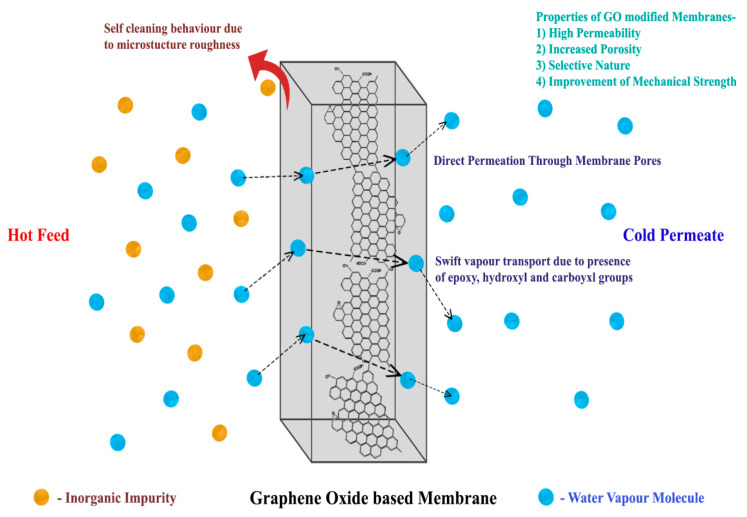
Property enhancement in MD membranes due to addition of graphene oxide nanoparticles.

Graphene oxide is a graphene derivative that has a novel architecture with the availability of various chemical functional groups on their surface that shows potential for selective separation processes. PVDF/GO membranes show higher fluxes due to the presence of higher porosity than pristine PVDF with favorable nanofiller–water interactions. They also tend to show enhanced mechanical strength as compared with pristine PVDF [104]. However, GO is inherently hydrophilic, which makes it necessary to functionalize it in order to achieve exceptional MD performance [105]. GO-ODA (octadecylamine-functionalized graphene oxide) membranes show high membrane flux due to an additional path of transport for vapor molecules [106]. The functionalization of GO by n-butylamine before introducing it into flat sheet PVDF membranes results in high mechanical strength due to the uniform crystal structure and superior interfacial interactions [106]. The blending of reduced GO into the PVDF membrane matrix resulted in enhanced MD performance due to morphological and functional modifications in the membrane [107]. Figure 9 gives a brief overview on the enhancement of membrane properties due to the addition of GO NPs.

#### 3.2.3. Quantum Dots (QDs)

**Figure 10:**
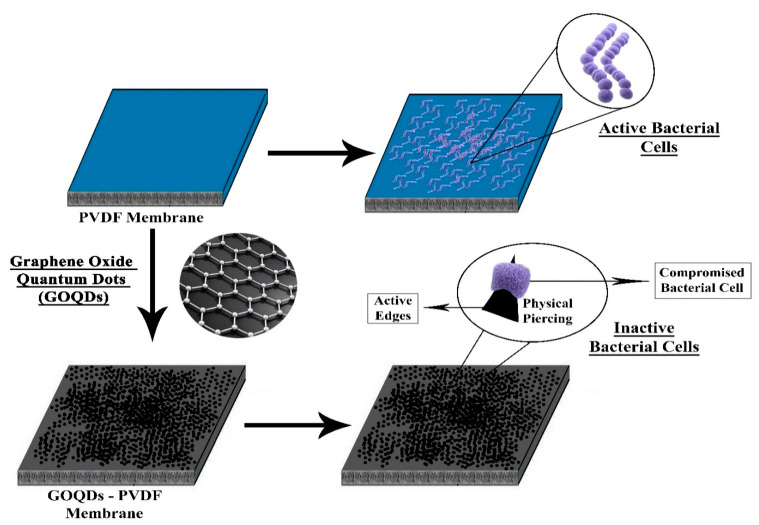
Demonstration of methodology to incorporate quantum dots (QDs) into a hydrophobic membrane.

Quantum dots have recently attracted much attention as an MD membrane modifier because of their physicochemical properties such as high biocompatibility, complete chemical inertness, ultra-small size, rich surface functional groups, and high anti-fouling ability. QDs are relatively more straightforward and inexpensive to incorporate on the membrane surface than other surface modifiers due to their ultra-small size and rich functionality. These modifiers can be selectively incorporated just on the substrate layer or the entire membrane depending upon the cost, material usage, and desired properties of the membrane. A QDs-enhanced MD membrane has shown significantly improved permeability, porosity, antibacterial, and anti-fouling capacity. In a study carried out by Jafari, A., graphene quantum dots (GQDs)-incorporated PVDF membranes, synthesized by the electrospinning method, were tested in an AGDM system. The addition of GQDs NPs caused a slight decline in the liquid entry pressure (LEPw) and contact angle, which can be related to the lower hydrophobicity of the membrane after modification because of the existence of hydrophilic functional groups in GQDs NPs. Overall, the obtained GQDs/PVDF electrospun membrane exhibited a more compact structure, acceptable contact angle, LEPw, and porosity. The modified membrane exhibited better anti-wetting properties than base PVDF membranes in a 60-h test. Further, the modified membrane GQD3P (0.25 wt% GQDs) achieved a higher flux value (17.6 L m^−2^ h^−1^) and a salt rejection of about 99.7%, compared with a pure PVDF membrane, which could achieve a membrane flux of 14.7 L m^−2^ h^−1^ and salt rejection rate of about 94.5%, which could be related to blockages caused due to a higher concentration of NPs, greater compactness of nanofibers ensuring lesser distance between the layers, and higher LEPw [84]. In another study, GOQDs were coated on the surface of the PVDF membrane through helium plasma-induced surface grafting of polyethylene glycol (PEG), followed by treatment with APTMS and covalent linkage between amine groups available on the PVDF membrane and carboxylic groups available on the GOQDs. The achieved GOQDs-modified PVDF membrane showed significantly enhanced water permeability, stability, antibacterial, and anti-biofouling properties. The improved anti-biofouling capacity was attributed to the presence of uniformly dispersed GOQDs NPs with a large number of active edges present on their surface, causing physical piercing of bacterial cells upon direct contact. The modified membrane also exhibited a lesser flux drop (23.4%) compared with the pristine PVDF membrane (65.7%) after 12 h [108]. Figure 10 illustrates the methodology and application of QDs into the MD membrane.

Based on a few reported studies where QDs have been utilized in an MD membrane, Table 3 represents the membrane performance in terms of membrane water flux, salt rejection rate, contact angle, and other applications. In short, it can be concluded that QDs-doped membranes seem to be efficient for improved anti-biofouling as well as long-term MD performance.

### 3.3. Metal Organic Framework (MOFs)

**Figure 11:**
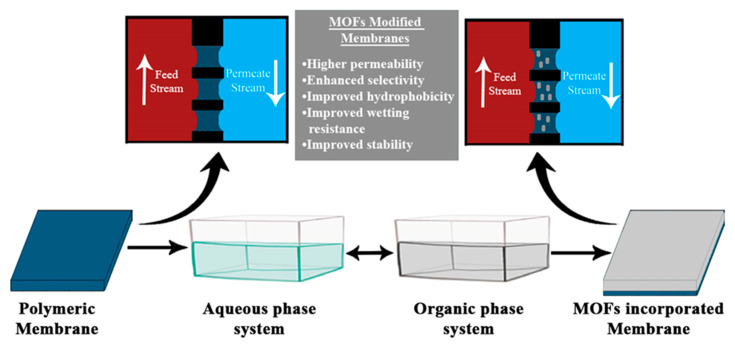
Incorporation of metal organic frameworks (MOFs) by interfacial polymerization utilized in the MD process.

In recent decades, membrane-based separation methods have attracted a lot of attention due to their applicability in a variety of methods for water and wastewater purification. As earlier mentioned, the MD method is reliable, flexible, and energy-efficient; however, the existence of inorganic and organic pollutants in water makes these methods highly prone to fouling and wetting that reduces the selectivity and overall efficiency of the membrane [112]. Therefore, in an effort to counter the aforementioned concerns, membranes are coated, impregnated, or mixed with nanomaterials, oxides of graphene, or metal oxide frameworks (MOFs) [113]. All these materials notably enhanced the hydrophobicity, selectivity, and performance of membranes in MD operations [113]. Conversely, MOFs have received substantial attention in MD operations owing to their desired physio-chemical characteristics like high surface area, porosity, and intensive capacity to combine inorganic metal centers with organic linkers by coordinate bonds without altering the framework [114,115]. Briefly, MOFs are hybrid inorganic-centered organic microporous crystalline structures. Although the applicability of MOFs is widespread, these materials are thermodynamically unstable. In recent investigations, MOFs with clustered centers of aluminum (Al), zirconium (Zr), and iron (Fe) exhibited stable characteristics for water treatment processes [116]. Prominently, till date, MOFs-incorporated membranes are only used for DCMD and VMD [117]. Figure 11 indicates the advantageous features of MOFs-doped MD and the process involved in the incorporation of MOFs in polymeric membranes.

As per the few reports, the incorporation of various MOFs reverts the membrane hydrophobicity to hydrophilicity, hence improving its wetting resistance and anti-fouling properties. The wettability of the membrane can be studied by determining its LEPw value. An increase in the LEPw value can be linked to the reduced pore size and increased hydrophobicity of the membrane, hence enhancing its wetting resistance. In a recent study, the LEPw value of an MOF-modified MD membrane was significantly raised to 3 bar, owing to the growth of hydrophobic floss on the surface [116]. In addition to that, MOFs-enhanced polymeric membranes also exhibited higher permeability and selectivity [118]. A summary of MOFs-doped polymeric membranes for MD processes is shown in Table 4.

## 4. Nanomaterials for Fouling Control in MD Process

Mitigating membrane fouling and surface wetting are amongst the most prominent areas of research in MD studies. The main aim while fabricating membranes for long-term operations is to design anti-wetting and superhydrophobic surfaces. The wetting resistance of the surface is controlled by the surface free energy of the membrane or membrane geometry. Thus, in order to minimize surface wettability, the two most commonly used approaches are (1) generating a hierarchal structure with micro/nano roughness or (2) lowering the surface free energy for the membrane by chemical modifications [121]. Altering the surface chemistry to generate membranes with low surface free energy alters membrane–liquid interactions, while fabricating hierarchal structures creates air pockets that help in achieving anti-wetting behavior. The superhydrophobic layer also shows anti-wetting ability against feed solutions possessing components having low surface tension that can quickly enter the pores of the membrane and thus contribute to pore wetting and fouling. Furthermore, these membranes tend to have higher permeability, thus improving membrane performance [122]. In this context, nanomaterials have been widely used to achieve superior liquid repellency, thus tackling the problem of membrane fouling and pore wetting [123]. Nanomaterial modifications onto electrospun nanofibrous membranes (ENMs) are often used to generate a hierarchal surface with several air pockets to repel water droplets and achieve the lotus leaf effect [124]. Inorganic metalloid and metal oxides such as titanium dioxide and silicon dioxide are extensively used to generate superhydrophobic membranes for MD application. TiO_2_ and SiO_2_ have an abundant amount of hydroxyl surface groups present on their surface that makes it easy to functionalize them with fluoroalkylsilanes and fabricate multifunctional membranes [125,126]. Application of carbon-based nanomaterials in membrane fabrication has enabled achieving robust anti-wetting surfaces owing to their excellent physicochemical characteristics [127,128]. The incorporation of MOFs results in an increment of the surface roughness of the membrane and thus enhances the anti-wetting behavior. The effects of anti-wetting behavior due to the incorporation of NPs are showcased in Table 5.

## 5. Current Challenges and Future Outlook

Membrane modifications utilizing nanomaterials, such as NPs, nanotubes, nano-composites, and organic metal frameworks, face challenges that prove futile for the membrane as well as for the overall process [1,135]. The most prominent issues are discussed as follows:

1. Efficient synthesis methods: The use of nanomaterials poses many barriers to successful membrane modification, such as membrane pore blocking and non-uniform dispersal of membrane nanomaterials, among others [1,136]. Hence, it is important to test and devise an efficient membrane synthesis method according to the requirements for successful membrane modification. Otherwise, the entire process may fail because the membranes will not be efficient and successful in their position;
2. Appropriate integration of the materials: The nanomaterials used to modify the membranes must adhere or embed in the membranes appropriately; otherwise, they may leach out over time [136]. The different commonly used nanomaterials, such as CNTs and metal oxide NPs, among others, could be linked to the membranes via different groups, like -OH, present on their surface with the functional groups present in the membrane matrix via hydrogen or covalent bonds. This will not only make the membrane effective and efficient but also enhance its stability and life;
3. Stability of the fillers: The stability of the filler material is very important as it defines the overall membrane effectiveness and efficiency [135,136,137]. They must be firmly embedded with the aid of various bonds in the membrane matrix. The firmly embedded filler material in the membrane satisfies that while in use, the filler material will not disintegrate from the membrane. The membrane can, therefore, be utilized for longer periods of time without losing its structural and functional integrity. However, presently much work is needed for developing such filler materials as well as synthesis methods that approve the stability of the fillers to cent percent;
4. Conservation of the functional properties: It is very important to conserve the required functional attributes of membranes for which they are sought. Currently, however, there is a definite loss of functional characteristics during membrane synthesis, meaning that the membranes cannot show the theoretical extent of their functional attributes. In addition, there is a loss of functionality over time during membrane operations. Therefore, there is a requirement of a sustainable synthesis method that is capable of withholding the functional capacity of the membranes to the best;
5. Membrane strength: The filler materials, sometimes instead of increasing the strength of the membrane, make them weak and brittle. Therefore, this aspect should also be studied carefully before using any filler for the modification of membranes;
6. Membrane fouling: Fouling is one of the most important factors in membrane science that inhibits the use of membranes in large-scale applications. However, there are lots of studies carried out specifically to eradicate this single problem, but it still persists [2]. Therefore, there is a need to tackle this problem appropriately for better employment of membranes for large-scale applications.

The recently reported literature based on the incorporation of nanomaterials suggested a few prominent future aspects which can be explored, such as:

(a) Risk of particle aggregation while incorporating nanomaterials onto the membrane;
(b) Utilization of cheap nanomaterials in order to reduce the overall cost;
(c) Rougher membrane surface may enhance membrane fouling, which must be optimized;
(d) Risk of peeling off or particle washout after a long-term experiment.

**Figure 12:**
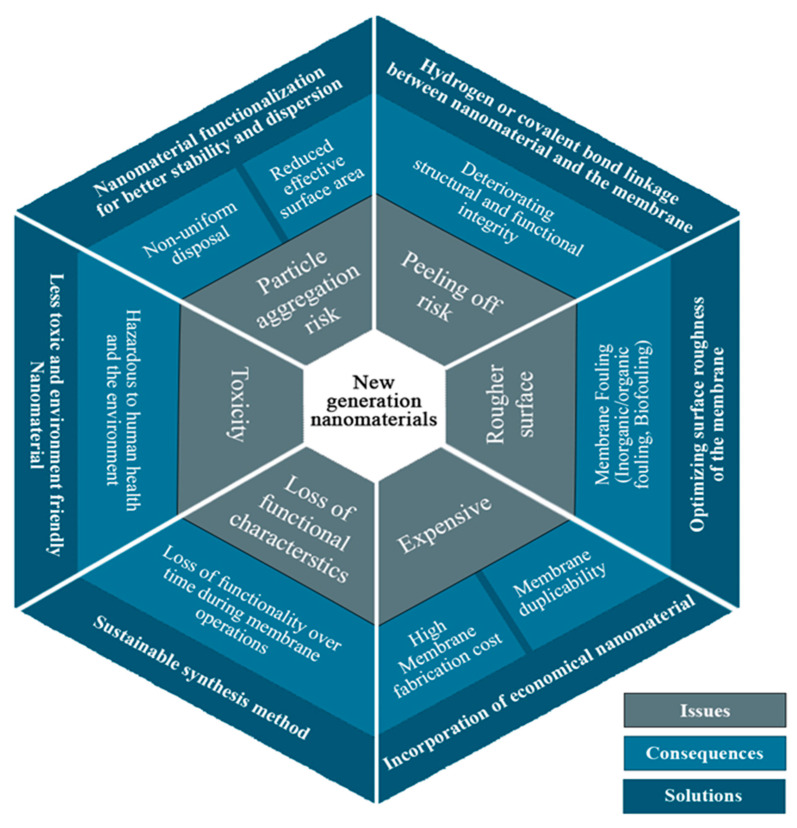
Summarization of serious issues related to the manufacture of nanomaterial-based polymeric MD membranes.

Figure 12 summarizes the serious issues in the manufacture of nanomaterial-based polymeric MD membranes, which need high attention and must be systematically discussed to attain improved MD membrane performance and scalability.

Thus, extensive research must be encouraged for the fabrication of superhydrophobic and anti-wetting MD membranes via the incorporation of new generation nanomaterials as inadequate suppliers are available. In addition to that, collaborative research work is highly needed between academia and industrial organizations as these membranes need to be commercialized at the industrial scale.

## 6. Concluding Remarks

The main goal of this review was to study the new generation nanomaterials currently being used for the development of state-of-the-art MD membranes. In MD research, nanomaterials such as TiO_2_, SiO_2_, MOFs, CNTs, and graphene and its derivatives were evaluated from a holistic perspective as additives. According to extensive literature studies, the introduction of NPs generally improves the superhydrophobic property of the membrane, reduces the fouling tendency, increases the porosity of the membrane, and improves the mechanical and chemical stability, which ensures the production of a superior MD membrane class. Improvement of these properties enables the fabrication of robust anti-wetting surfaces for long-term MD operations. The methods of incorporation of NPs as a part of the membrane fabrication process are also briefly discussed. Even though using NPs as filler materials results in synergistic effects by imparting novel structural and functional characteristics, several problems such as high fabrication costs, scalability, hazardous nature, and arduous methods of designing are commonly observed. Therefore, in order to extend the work done in small-scale laboratories to large-scale industries, it is desired that future researchers seek the discovery of more viable and successful solutions. Thus, nanomaterial-based membrane engineering is strongly encouraged to reduce the research gap towards developing a supreme class of MD membranes.

## Figures and Tables

**Table 1:** Overview of desired membrane characteristics for MD application.

Criteria	Description	Desired Value	Ref.
**Liquid Entry Pressure (LEPw)**	LEPw is the pressure required for the liquid to overcome the forces of hydrophobicity and penetrate the pores of the membrane. It is desired for the external pressure to be less than LEPw to allow the proper functioning of an MD system. It is expressed using LEPw = $\frac{-2B\gamma l\;Cos\theta }{r_{max}}$ *B* is a geometric pore coefficient (equal to 1 for cylindrical pores), *γ* is liquid surface tension, *θ* is the contact angle, and *r_max_* is the maximum pore radius.	LEPw > 250 kPa	[47]
**Mean Pore Size and Pore Size distribution**	Permeability depends on the mean pore size. Larger pore size allows a greater area for mass transfer, thereby increasing the overall membrane flux. However, increasing the pore size reduces the LEPw, hence it is necessary to find the optimum pore size to find a balance between LEPw and membrane permeability Pore size distribution (PSD) indicates the variation in pore size and hence the variation in mass transfer and heat transfer mechanism with it, throughout the surface. Overall, PSD has a minimal effect on MD performance.	Mean Pore Size = 100 nm–1 μm	[48]
**Hydrophobicity**	Hydrophobicity is a crucial aspect when the fabrication material for the membrane is chosen. It is quantified with respect to the contact angle (θ_CA_) of water between the liquid surface and the membrane surface.	θ_CA_ > 90° (Hydrophobic) θ_CA_ > 150° (Superhydrophobic)	[49]
**Chemical Resistance**	The material used for membrane fabrication must show good resistance to chemicals (acids, bases, surfactants) to prevent membrane fouling and consequent wetting.	-	[50]
**Thermal Conductivity**	Membranes are desired to have a low thermal conductivity in the MD operation as it directly relates to the heat transfer through the membrane. Increased heat transfer would affect the vapor pressure equilibrium, thereby reducing the transmembrane flux.	0.1–0.5 W m^−1^ K^−1^ is the range commonly observed in the literature	[47]
**Membrane Thickness**	Optimum membrane thickness is required as it has major effects on the thermal conductivity and the membrane flux. Even though reducing the membrane thickness increases the membrane flux, it severely reduces the thermal resistance.	30–60 μm	[51]
**Membrane Porosity** (ε)	Membrane porosity refers to the fraction of voids present in the membrane to the total volume of the membrane. Increasing membrane porosity improves the flux transfer as well as the thermal resistance of the membrane; however, it is achieved at the expense of the mechanical strength of the membrane. $\varepsilon =1-\;\frac{\rho_{m}}{\rho_{pol}}$ where *ρ_m_* and *ρ_pol_* are the densities of the membrane and polymer, respectively	ɛ > 80%	[4]
**Tortuosity** (τ)	The irregularities of membrane pores from the ideal cylindrical pores are quantified by tortuosity. Highly tortuous structures result in lower flux as the vapor molecules suffer deviation from the direct path of transport. $\tau =\frac{{\left(2-\varepsilon \right)}^{2}}{\varepsilon }$	1.1–3.9 has been observed for most MD systems	[52]
**Tensile Strength**	The membrane material should possess adequate tensile strength to be assembled and fixed in membrane modules as the operational pressures are much less compared with RO, UF, and MF.	3.4–54.9 MPa is commonly observed for most MD membranes.	[53]
**Sliding Angle**	Sliding angle is another criterion along with contact angle used to measure surface hydrophobicity. Lower sliding angle indicates higher hydrophobicity as the water droplets do not adhere to the membrane surface.	<10°	[54]
**Surface Roughness**	Microstructure roughness results in the formation of air pockets which results in improving membrane hydrophobicity.	Optimized surface roughness provides air layers which ultimately leads to higher hydrophobicity	[55]

**Table 2:** State-of-the-art of nanomaterial-based MD membranes for improved desalination performance.

Membrane	Nanomaterials	MD Type	Category	Pore Size (µm)	Flux (L m^−2^ h^−1^)	Contact Angle (°)	Ref.
**PVDF-HFP/Si(NPs)**	Silica	DCMD	Metalloid	1.28	48.6	>150°	[75]
**PVDF- TiO_2_(NPs)**	Titanium dioxide	DCMD	Metal oxide	0.4 ± 0.05	2.5	140°	[76]
**FTCS-TiO_2_-PVDF**	Titanium dioxide	DCMD	Metal oxide	0.45	30	163 ± 3°	[77]
**S-PVDF-20**	Silver	UVMD	Metallic	0.475	2.1	148 ± 2.1°	[78]
**OMNI** **(ZnO-GF)**	Zinc oxide	DCMD	Metal oxide	0.4	11.4 ± 0.9	152.8 ± 1°	[79]
**FAS-SiNPs-SFM**	Silica	DCMD	Metalloid	0.85	21.9 ± 1.2	-	[80]
**PVDF-SiO_2_(NPs)**	Silicon dioxide	VMD	Metalloid	0.14	2.8	94°	[42]
**PVDF-Al_2_O_3_(NPs)**	Aluminium oxide	AGMD	Metal oxide	0.370	20	153°	[43]
**PVDF-M-CNT**	Carbon nanotubes	DCMD	Carbon	0.14	35.1 ± 0.7	-	[81]
**PVDF-CNTs**	Carbon nanotubes	VMD	Carbon	0.20	28.5	159°	[82]
**GNP-Polyethene**	Graphene	DCMD	Carbon	0.15	16.7	123°	[83]
**GQDs-PVDF**	Graphene quantum dots	AGMD	Quantum dots	0.0049	17.6	>125°	[84]
**MWCNTs/** **SiO_2_-PVDF**	Multi-walled carbon nanotubes and silicon dioxide	VMD	Carbon	0.09	2.5	91 ± 2.1°	[85]

**Table 3:** An overview of feasible methodologies to cast quantum dots (QDs)-doped hydrophobic polymeric membranes and the overall outcome.

Membrane	QD Type	Contact Angle (°)	Application	Ref.
**(GQDs)/PVDF**	Graphene quantum dots	>125°	Higher flux Improved salt rejection	[84]
**C18-CQDs**	Carbon quantum dots	152.2 ± 1.25°	High water vapor permeability High salt rejection rate (up to 99%) Highly efficient oil–water separation (up to 99%)	[109]
**GOQDs-PVDF**	Graphene oxide quantum dots	34.3 ± 2.6°	Improved antibacterial and anti-biofouling properties Lower flux drop	[108]
**GOQDs-PSF**	Graphene oxide quantum dots	65°	Enhanced permeability Enhanced flux Improved anti-fouling properties	[110]
**PVAx-GOQD300**	Graphene oxide quantum dots	53.8 ± 0.1°	Improved stability	[111]

**Table 4:** A brief summary of MOFs-doped MD membranes used in various MD modules.

Membrane	MOFs Type	Membrane Type	MD Module	Contact Angle (°)	LEPw (KPa)	Ref.
**MOF-functionalized alumina tub**	NH2-MIL-53(Al)	Tubular	(VMD) Vacuum membrane distillation	-	300	[116]
**ZIF-8/PDMS**	ZIF-8	Hollow fiber	(DCMD) Direct contact membrane distillation	130°	-	[119]
**(Iron-BTC)/PVDF**	Iron-BTC	Flat sheet membrane	(DCMD) Direct contact membrane distillation	138.06 ± 2.18°	82.73	[118]
**AlFu MOF/PVDF**	AlFu	Hollow fiber	(DCMD) Direct contact membrane distillation	>100°	-	[117]
**MOFs/SiO_2_-PVDF**	MOF-808	Flat sheet membrane	(DCMD) Direct contact membrane distillation	140.8°	86.2 ± 3.2	[120]

**Table 5:** Effect of nanoparticle addition on anti-wetting and anti-fouling behavior.

Base Polymer	Nanomaterial	Mode of Fabrication	Configuration	Water Contact Angle (°)	LEPw (kPa)	Mean Pore Diameter (µm)	Performance Characteristics	Ref.
**PVDF**	TiO_2_	Phase inversion	DCMD	112 ± 1.4	64 ± 3	0.44 ± 0.02	Self-cleaning effects under UV light with a higher flux recovery ratio as compared with unmodified PVDF	[76]
**PVDF-co-HPF**	FTES-functionalized TiO_2_	Electrospinning followed by electrospraying to coat TiO_2_ NPs	DCMD	157 ± 1.6	-	0.52	Mitigation of membrane fouling with regenerative abilities for long-term performance	[129]
**PVDF-HPF**	Si	Electrospinning	DCMD	> 150	76.4	1.70	Total of 99.99% salt rejection over 240 h of desalination experiments showing long-term permeability	[130]
**PVDF**	PFOTS-modified SiO_2_	Immersion deposition	DCMD	161.5	-	0.2 ± 0.01	Steady operation over 156 h with feed consisting of NaCl (100 g/L), CaCl_2_ (1.26 g/L), and humic Acid (10 mg/L)	[131]
**PVDF**	FAS-modified SiO_2_ (8% wt)	Electrospinning	VMD	160.5 ± 2.3	195	0.26 ± 0.02	Pore wetting prevented due to high LEPw value showing a permeate flux of around 30 l. m^−2^h^−1^	[88]
**PVDF**	Aluminum fumarate MOF (1%)	Dry-jet wet phase inversion	DCMD	> 100	>200	0.3	Stable salt rejection of 99.9% for 3.5 wt% NaCl solution over 50 h of operation	[117]
**PVDF**	ZnO NPs modified by silane and coupled with ZIF-8 crystal	Phase inversion	DCMD	70	100	-	Modified membrane did not have a definite trend for permeate flux due to blocking of pores possibly due to wetting and scaling	[132]
**PVDF**	Triple-layered membrane with SiO_2_ (hydrophobic) blended in PVDF, PAN-MOFs, SiO_2_ (hydrophilic) blended in PVDF	Electrospinning	DCMD	140.8 ± 9.9	86.2	0.31−1.22	Hydrophobic SiO_2_-NPs increase the permeate flux while MOFs increase the pore size of the middle layer that contributed to superior DCMD performance for 5 h with low permeate conductivity	[120]
**PVDF**	CNTs	Electrospinning followed by spray gun to coat CNTs	VMD	159.3	188	0.2	Even though the membrane had a stable performance for 14 h of operation, increasing the CNT loading beyond a point did not improve pure water flux due to the increased thickness of the membrane	[133]
**PTFE**	GO	Dropwise coating of PVDF-GO onto flat sheet PTFE membranes	DCMD	75 ± 2	-	0.2	Hydrophilic properties of GO improve mass transfer coefficient, thus improving membrane flux with stable performance for 60 days of operation	[134]
